# Comparative Anatomy of Mitral and Tricuspid Valve: What Can the Interventionlist Learn From the Surgeon

**DOI:** 10.3389/fcvm.2018.00080

**Published:** 2018-06-29

**Authors:** Alberto Pozzoli, Michel Zuber, Mark Reisman, Francesco Maisano, Maurizio Taramasso

**Affiliations:** ^1^Heart Valve Clinic, University Hospital of Zurich, University of Zurich, Zurich, Switzerland; ^2^Division of Cardiology, Department of Medicine, University of Washington, Seattle, WA, United States

**Keywords:** tricuspid valve, mitral valve, transcatheter therapies, comparative anatomy, multimodality imaging

## Abstract

Transcatheter valve interventions on the mitral and tricuspid valves entail increasing complexity. Part of the knowledge that has been generated during the development of mitral devices can be transferred to the tricuspid valve (TV). However, a deeper understanding of the peculiar anatomy of the TV and of the right heart chambers, together with differences and similarities between the two valves, is fundamental. This report compares the anatomy of the mitral and tricuspid valves, and its inferences with regard to transcatheter treatments.

**Condensed abstract:**

This report explores anatomical similarities and differences between the mitral and the tricuspid valves, and their implications with regard to transcatheter treatments.

## Introduction

Transcatheter valve therapies deeply changed the treatment of heart valve disease over the last decade. Shifting from aortic valve interventions (TAVI), more reproducible and with less anatomical variables, toward the AV valves entails increasing complexity and deeper knowledge of these two valves. The development of transcatheter mitral valve (MV) therapies was much more slower, mainly due to the structure and complexity of the MV apparatus and its pathology. With the development of transcatheter tricuspid valve (TV) therapies, interventionists are dealing with an even more stimulating anatomical scenario. Part of the knowledge that has been generated during the development of mitral devices can be transferred to the TV. Therefore, a deep knowledge of the tricuspid anatomy and of the right heart chambers, comparing the differences between the two AV valves, becomes fundamental (1). In this report, the anatomical similarities and differences between mitral and tricuspid apparatus, and their impact (effect) with regard to transcatheter treatments (Table 1) will be addressed.

**Table 1:** Similarities and anatomical differences between mitral and tricuspid valve apparatus, and their implications with regard to transcatheter treatments.

**Left ATRIUM and LAA**	**Right ATRIUM and RAA**	**Interventional considerations**
Thicker walls than the RA Smooth atrial cavity Long and narrow trabeculated LAA Presence of PVs orifices	Thinner and more distensible walls Presence of Crista Terminalis Wide and blunted RAA Presence of SVC, IVC, and CS orifices	RA is reached either from SVC or IVC LA is reached mostly through the septum and provides support to device delivery system Higher chance of LAA perforation
**Mitral ANNULUS**	**Tricuspid ANNULUS**	**Interventional considerations**
Attached to 2 fibrous trigones (AL-PM) Saddle-shaped in systole Fibrous structure is thick Contiguity with the His bundle (PM commissure), the coronary sinus and the Cx artery (posterolateral region)	Attached to only 1trigone (PM) Easily distensible with thinner and almost virtual fibrous structures Largest orifice of all valves (7–9 cm) Contiguity with the Koch triangle, RCA (anteroposterior) and aortic cusps	TV annular procedures are most prone to injury the RCA (longer course), the AV node and His bundle (AV block) and the aortic cusps (AR) TV imaging guidance is more challenging (“TEE-unfriendly,” ICE frequently needed) Complete obliteration of EROA and valve sealing is cumbersome in tricuspid position
**Mitral LEAFLETS and COMMISSURES**	**Tricuspid LEAFLETS and COMMISSURES**	**Interventional considerations**
2 leaflets (A-P) and 2 commissures Thicker and more resistant than TL	3 leaflets (A-P-S) and 3 commissures Thinner, translucent and more fragile	Higher chance to damage or tear the TV leaflets
**Mitral CHORDAE TENDINAE**	**Tricuspid CHORDAE TENDINAE**	**Interventional considerations**
Thicker and more resistant. Bifurcated/trifurcated at the free edge Extend directly from the heads of PMs	Thinner and more fragile Single attachment at the free edge Originating from various level of PMs and can attach directly to the RV wall	High chance of entrapment and impinging the commissural chordae, once the valve is crossed Higher risk in the AS commissural region of the TV
**Mitral PAPILLARY MUSCLES**	**Tricuspid PAPILLARY MUSCLES**	**Interventional considerations**
2 papillary muscles (AL-PM) Single bulky or multiple heads No PMs is attached to the septum	3 papillary muscles (ANT dominant- POST-SEPT, multiple and thinner heads) Can originate from the septum	Higher chance of catheter entrapment, especially in the antero-septal commissural region
**Left VENTRICLE and LVOT**	**Right VENTRICLE and RVOT**	**Interventional considerations**
Thicker walls than the RV (3:1) Absence of Moderator Band MV is in continuity with the AV through the mitro-aortic curtain cohoctic cavity	Thinner and more distensible walls (1:3) Presence of Moderator Band TV and PV are widely separated Crescentic cavity	Increased risk of LVOTO during TMVR Risk of RVOTO is negligible/absent RV transapical access suboptimal for coaxiality and potentially risky in thin,dilated RV

*RA, right atrium; LA, left atrium; RAA, right atrial appendage; LAA, left atrial appendage; PVs, pulmonary veins; MV, mitral valve; TV, tricuspid valve; PV pulmonary valve; SVC, superior vena cava; IVC, inferior vena cava; CS, coronary sinus; AL, anterolateral; PM, posteromedial; A, anterior; P, posterior; S, septal; AS, anteroseptal; Cx, circumflex artery; RCA, right coronary artery; AR, aortic regurgitation; TEE, transesophageal echo; ICE, intracardiac echocardiography; EROA, effective regurgitant orifice area; PMs, papillary muscles; LVOTO, left ventricular outflow tract obstruction; RVOTO, right ventricular outflow tract obstruction; TMVR, transcatheter mitral valve replacement; RV, right ventricle; AV, aortic valve. This table has been adapted from Taramasso et al. (1)*.

## Left atrium, right atrium, and interventional access

### Anatomical description

#### Left atrium

**Figure 1:**
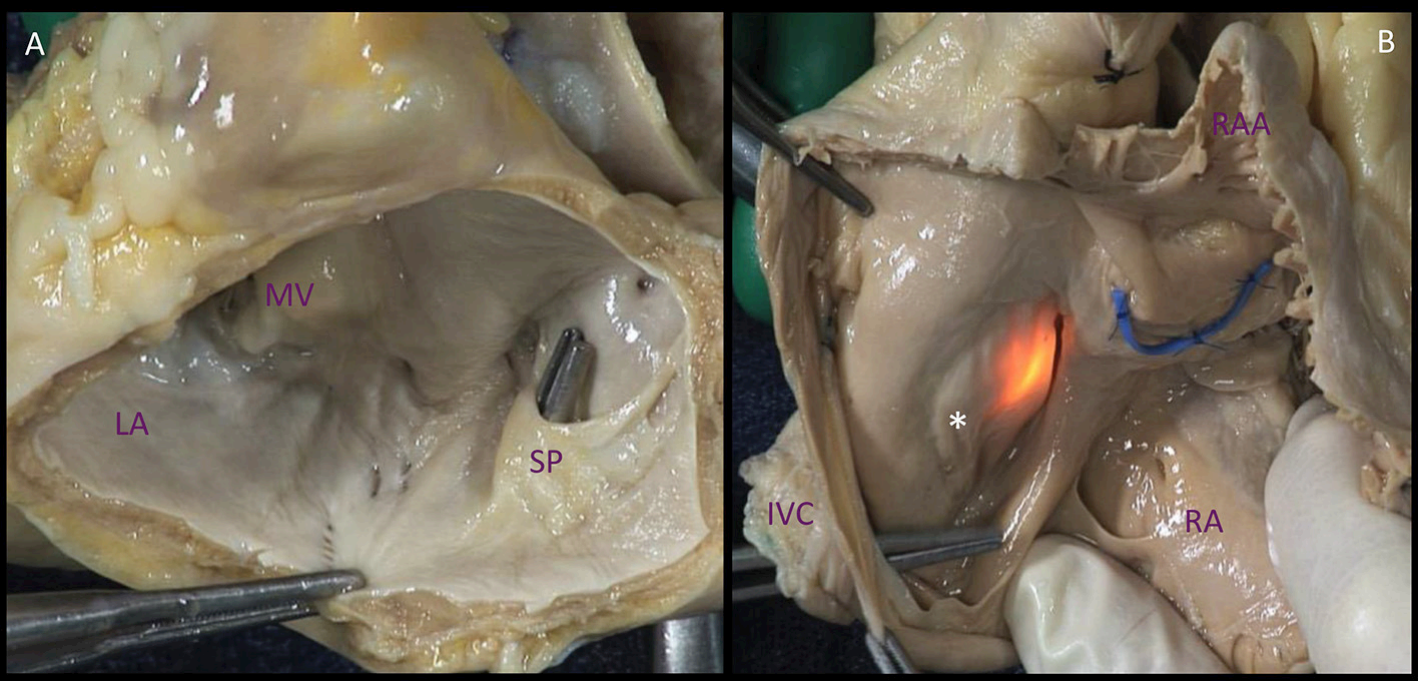
Comparison of the atrial septal surfaces. **(A)**. The septal surface of the left atrium is characterized by the flap valve of the fossa ovalis (septum primum), in contrast to the limbus of the fossa ovalis (^*^) present on the right atrioseptal surface **(B)**. The source of light indicates the fossa ovalis, the blue band depicts the position of the aortic non-coronary sinus. LA, left atrium; MV, mitral valve; SP. septum primum; IVC, inferior vena cava; RA, right atrium; RAA, right atrial appendage.

The left atrium (LA) is the cardiac chamber that normally receives pulmonary venous drainage from the four pulmonary veins. Its septal surface is characterized by the flap valve of the fossa ovalis (septum primum), in contrast to the limbus (septus secundum) of the fossa ovalis present on the right atrioseptal surface (Figure 1).

#### Right atrium

The right atrium (RA) consists of a curved posterior groove continuous with the superior and inferior venae cavae, a flat interatrial septum, a trabeculated dome, and the TV. In comparison to the LA, the RA has thinner walls and dilates more easily given the same degree of pressure overload.

### Interventional considerations

Different interventional accesses to the LA have been adopted, including direct transatrial, transapical, transarterial retrograde, and transseptal.

Transatrial and transarterial retrograde routes are currently used only in really specific situations and have almost been abandoned.

The transseptal access, through the inferior vena cava favored by the crista dividends, is currently the preferred route for most transcatheter MV repair techniques and its usage is quickly increasing for mitral valve-in-valve and valve-in-ring procedures, since it showed superior safety compared to the apical one (2, 3). Transapical access is the most used approach for native MV replacement and for transcatheter neo-chordal implantation, and it will be discussed below in the section describing the ventricles.

Transseptal catheterization is a safe and well-known approach to the LA and, therefore, to the MV. As an example, transseptal route is used for MitraClip (Abbott Vascular, USA) and for direct annuloplasty with the Cardioband device (Edwards Lifescience, USA). Both of the devices are delivered through a big (24F) steerable guiding catheter, which allows the operator to reach the anatomical therapeutic target with a high level of precision, required to ensure safety and efficacy.

Therefore, to guarantee the needed precision, the location of the transseptal puncture is essential, since a specific therapeutic target could be extremely challenging or even impossible to reach with a proper trajectory, if the puncture is performed in a wrong location. To this aim, operators should be familiar with the anatomical structures in proximity to the interatrial septum: in case of a too anterior or too posterior puncture, the ascending aorta or the posterior LA wall, respectively, can be punctured and injured. Procedural imaging with TEE is the key to perform precise and safe transseptal puncture in complex structural interventions. Once the guiding catheter has been introduced in the LA through the interatrial septum, the septum gives the catheter itself adequate support and optimal stabilization, which allow the operators a really controlled and predictable steering of the guiding catheter.

Navigation in the LA can be extremely challenging and potentially dangerous in presence of a small LA, due to reduced degrees of movements, with increased risk of perforation, impingement, and bleeding. The structures at higher risk are the LAA and the pulmonary veins. In particular, the LAA is located anteriorly to the fossa ovalis, and it is easy to reach when crossing the septum if the atrium is not enlarged.

Similarly to the mitral valve, the tricuspid is commonly approached anterogradely. Currently, the most used approach is the transfemoral one through the inferior vena cava (IVC) (MitraClip, Cardioband, TriCinch), whereas some devices are delivered through a transjugular approach (Trialign, Forma).

Since the TV is approached directly without transseptal puncture, the support provided by the interatrial septum to the catheter in transseptal MV procedures is missing, resulting in a complete lack of stabilization. The absence of the septal support results in diving into the right ventricle (RV) and lack of coaxiality. This represents a major issue, making navigation in the RA more challenging and less controlled.

## Mitral and tricuspid annuli

### Anatomical description

#### The mitral annulus

**Figure 2:**
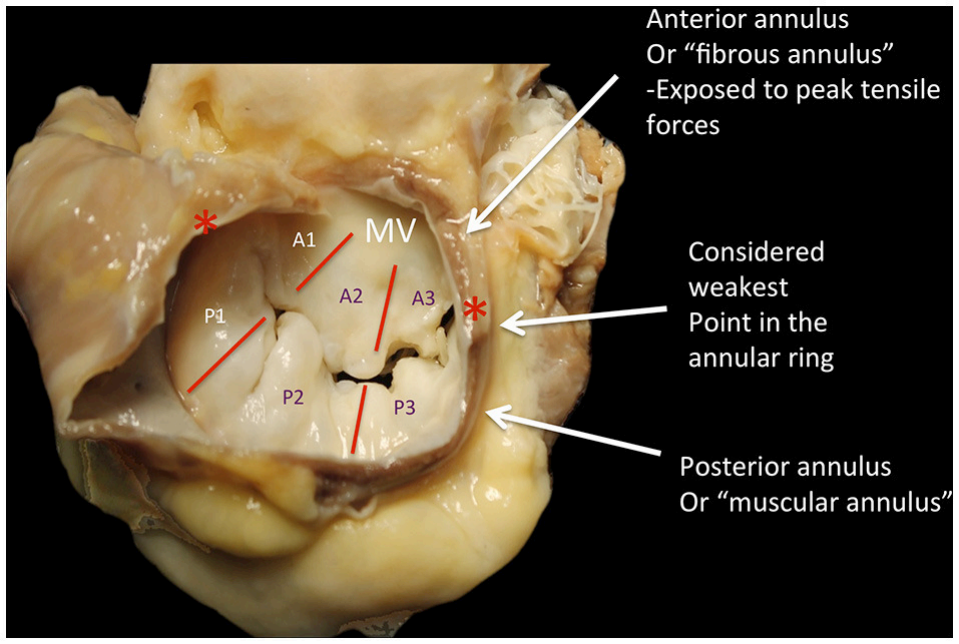
The annulus fibrosus of the mitral is a discontinuous band of connective tissue that exists only in some parts of the attachment of the posterior leaflet, between the two fibrous trigones (red asterisks). The annulus does not exist at the attachment of the anterior leaflet, because the leaflet tissue is continuous with the aorto-mitral curtain that extends from the aortic valve annulus to the base of the anterior leaflet. During the diastole, the shape is grossy circular; during systole it has a saddle shape, with antero-septal diameter significantly smaller than the intercommissural diameter. The anterior leaflet is primarily related to the left ventricular outflow tract via the aorto-mitral curtain, whereas the posterior one is related to the muscular parietal base of the left ventricle. The systolic reduction of the mitral orifice is around 25%, due to the contraction of the base of the heart and the displacement of the aorto-mitral curtain toward the center of the orifice. P1-P3, posterior scallops of the mitral leaflet; A1-A3, anterior scallops; MV, mitral valve; Courtesy of Dr. M. Reisman, University of Washington.

The mitral annulus is reinforced at each extremity of the base of the anterior leaflet, by two dense triangular fibrous structures: the antero-lateral (or left) and the postero-medial (or right) fibrous trigones (Figure 2) (2). Very important, the MV annulus has a 3D saddle-shape configuration and its shape varies through the cardiac cycle (3). Four anatomical structures close to the mitral annulus are at risk of injury during interventional procedures:

- The circumflex artery, which runs posteriorly and could be injured, especially during annuloplasty;
- The coronary sinus, which skirts the attachment of the posterior leaflet;
- The bundle of His which is located near the right trigone (medial commissure);
- The non-coronary and left coronary aortic cusps which are in close relationship with the base of the anterior leaflet, the so-called mitro-aortic fibrous continuity (there is a 6–10 mm safety zone in this area).

#### The tricuspid annulus

**Figure 3:**
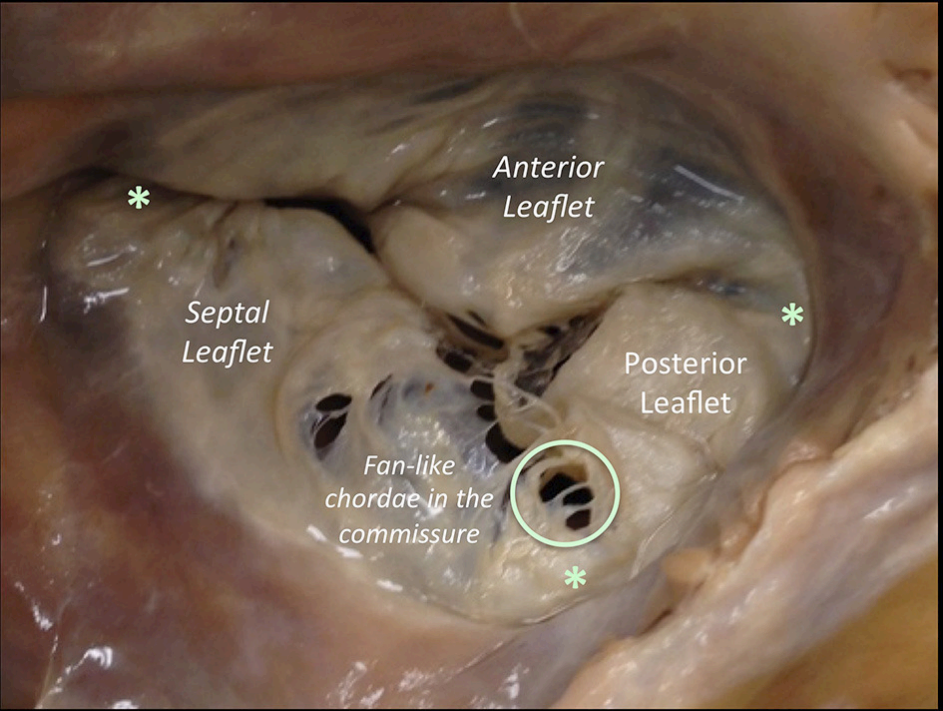
The anterior leaflet of the TV is larger than the posterior, which is larger than the septal leaflet. The anterior leaflet is primarily attached to the right ventricular outflow tract, the posterior to the muscular wall of the right ventricle and the septal one to the septum. The anterior has a semicircular shape and likewise the MV, it is divided into an atrial zone, regular, and thin, and into a distal zone, the zone of coaptation, slightly irregular and thicker than the proximal one. In comparison to the MV, few chordae are attached to the ventricular side of the leaflet. The posterior leaflet, slightly smaller than the anterior is divided in the same proximal and coaptation zones. The septal leaflet is the smaller and less mobile, and roughly semicircular and likewise the other two leaflets, it has a proximal and a coapting zone. The commissures of the TV are three and separate the three leaflets (red asterisk). They are small semilunar leaflets, attaching on the annulus and with a free edge attaching characteristics fan-like chordae. There is one variable to take into account: in comparison to the left, the septal attachment of the TV is at a more apical level than the septal attachment of the MV. As a result, a portion of the membranous interventricular septum separates the LV from the right atrium (this anatomical feature explains left ventricular to right atrial shunts diagnosed in congenital malformations). This image has been adapted from Taramasso et al. (1).

The right AV Junction delineates the change between the RA and the TV leaflets (Figure 3). Contrarily to the mitral one, the tricuspid annulus is tiny and difficult to identify and delimitate; annular calcifications are almost absent. In pathologic conditions, such as long lasting tricuspid regurgitation (TR), the TV annulus tends to become planar (4, 5).

**Figure 4:**
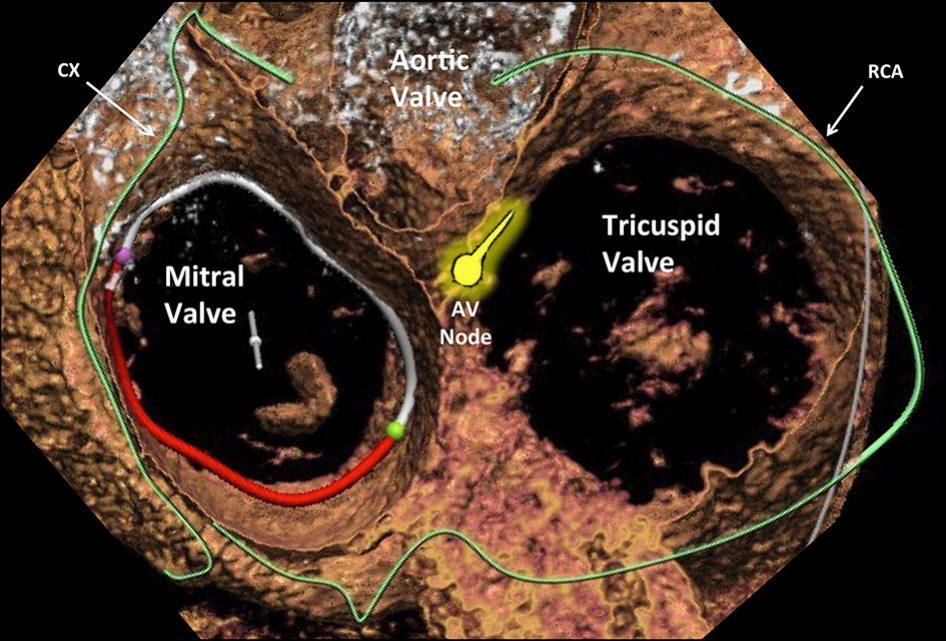
Cross-sectional view of the three heart valves seen from above with atria removed. Advanced editing of a computed tomography image performed with 3mensio (Pie Medical Imaging, Netherlands) software. The proximity of the atrioventricular valves (different area can be appreciated) to the coronary arteries that are located in the atrioventricular groove (the circumflex for the mitral and the right coronary artery for the tricuspid) represents a similar aspect to be taken into account. Cx, circumflex artery; AVN, atrioventricular node; RCA, right coronary artery.

Three anatomical structures close to the tricuspid annulus could be at risk of injury during interventional procedures:

- The non-coronary sinus of Valsalva, in particular the commissure between the non-coronary and the right coronary aortic cusp (especially in annuloplasty procedures);
- The bundle of His, which penetrates the central fibrous body and runs underneath the membranous septum 3–5 mm from the antero-septal commissure (the true landmark of His bundle) (Figure 4);
- The right coronary artery, the large single vessel coursing down the right AV groove and surrounding anteriorly the anterior TV leaflet.

### Interventional perspectives

**Figure 5:**
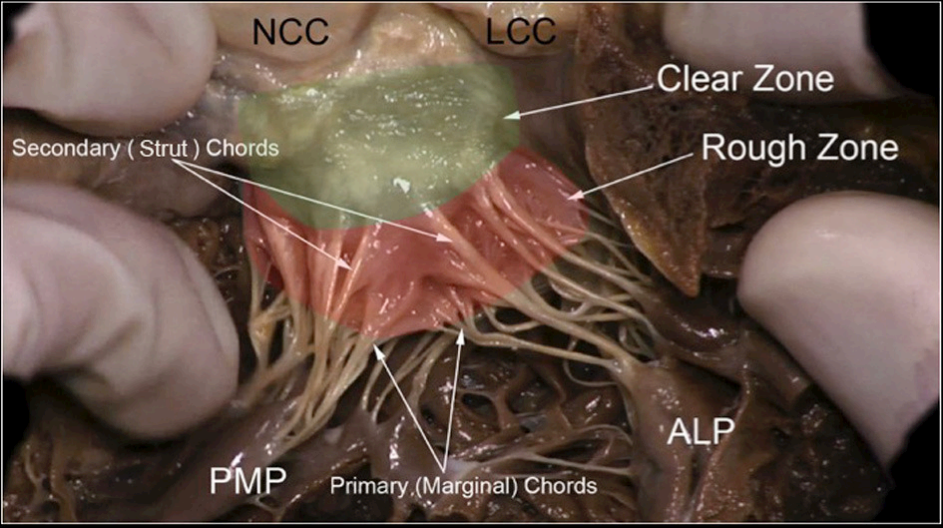
The leaflets of the MV are clearly divided into two regions, the atrial zone which is at the base of the leaflets (green clear zone), thin and translucent, and the zone of coaptation, which is the distal rough and irregular zone (red rough zone), where numerous thick chordae origin and attach the leaflets to the PMs. Three types of chordae tendineae can be described: tertiary chordae which origin normally directly from the LV and are attached to the base of the posterior leaflets and commissure. The secondary chordae (Strut) extend directly from the PM and are attached to the body of the leaflets, ventricular side. The primary chordae (marginal), the most represented and robust, are attached to the free margin of the leaflets and the space between them is never more than 3 mm. The attachment to the free margin is normally bifurcated or trifurcated. When they are considered upon their position, it is possible to recognize one thick and resistant ≪ main chorda ≫ attached to the ventricular surface of the leaflet which forms with the opposite main chord a kind of arcade, supporting the center part of the leaflet. The commissural chordae, attached to the commissural tissue, are trifurcated giving them the characteristic fan-like appearance. PMP, posterior mitral papillary; ALP, anterior leaflet papillary; NCC, non-coronary cusp; LCC, left coronary cusp. Courtesy of Dr. M. Reisman, University of Washington.

The more anterior location of the TV compared to the MV (which is much more “TEE-friendly”) makes intraprocedural TEE guidance particularly demanding in tricuspid procedure. In some circumstances, a combination of TEE, TTE and intracardiac echocardiography (ICE) is needed to obtain adequate imaging quality. The major interventional issue related to the TV compared to the MV is its larger orifice (Figure 5). If in normal conditions the TV area can already reach up to 9 cm^2^, this area will be much larger in the presence of functional TR, representing more than 90% of TR etiology. In such a condition, the regurgitant orifice area is often bigger than 1 cm^2^, i.e., more than double than in mitral position usually central and with a larger coaptation gap compared to MV. Therefore, a complete obliteration of the regurgitant area can be extremely cumbersome with the current repair devices. Similarly, it is easy to understand that also a replacement device has to be extremely big to cover the whole TV area. The large anatomy and the absence of annular calcifications are probably the two most important challenges to obtain sealing with a replacement device in TV position compared to the MV. The proximity of other cardiac structures has interventional implications in both mitral and tricuspid position. A peculiarity of the TV is the contiguity of the AV Node and His bundle (Figure 4), which is located in proximity of the septal TV annulus, close to the antero-septal commissure (the most common therapeutic target in MitraClip tricuspid procedures). In fact, an acute and complete AV Block (or even asystolia) can be induced just by the contact of any device with the His bundle, due to its compression.

Another important anatomical difference between MV and TV annuli from an interventional perspective is the different risk of coronary injury. The risk of coronary damage during interventional mitral or tricuspid procedures is mainly present in annuloplasty procedure (both direct and indirect), and it is highly dependent on the coronary anatomy and dominance of the specific patients (Figure 4). Every therapy addressing the tricuspid annulus in a direct way, especially for unpractised operators, implies an augmented risk of damaging the right coronary artery.

## Valve leaflets

### Anatomical description

#### The mitral leaflets

The MV comprises two leaflets, the anterior (or aortic) and the posterior (or mural), which are separated by two commissures (Figures 2, 5) and without the septal attachment. The valve leaflets are segmented into six sections: from P1 to P3 for the posterior and from A1 to A3 for the anterior. This classification has been useful in describing morphology observed during surgical operation (6), multiplane 2D TEE echocardiography and 3D echocardiography (7).

#### The tricuspid leaflets

**Figure 6:**
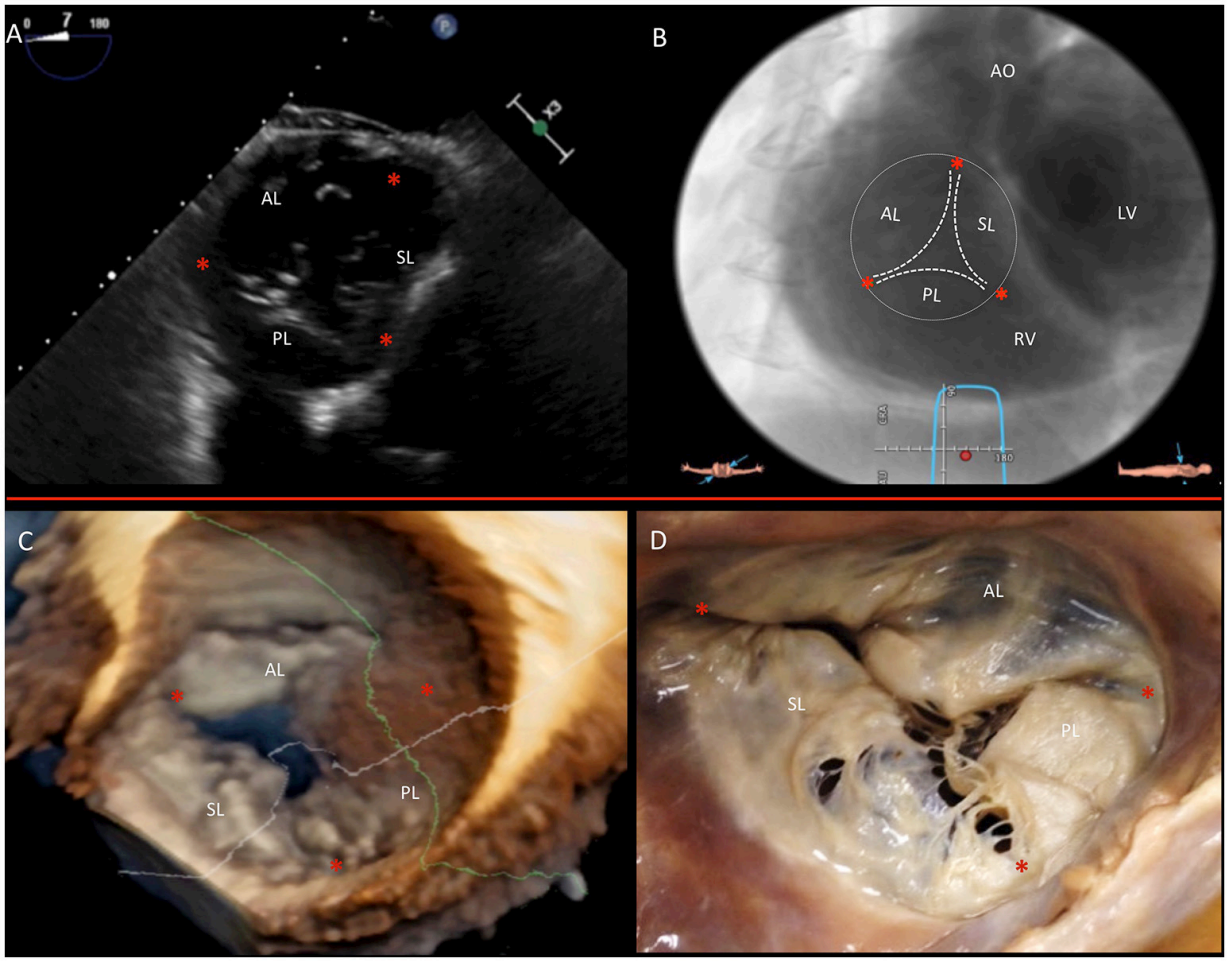
Multimodality imaging of the TV. **(A)** Deep transgastric 2D TEE view, displaying the TV in short axis from the ventricular side. **(B)** Same en face view derived from MSCT angiography. **(C)** Short-axis atrial view of the TV on 3D TEE and during surgery **(D)**. Red asterisks are the commissures. AL, anterior leaflet; LV, left ventricle; PL, posterior leaflet; RV, right ventricle; SL, septal leaflet. This image has been adapted from Taramasso et al. (1).

The TV comprises three leaflets: the anterior, the posterior and the septal, which are separated by three commissures. The septal leaflet is characteristic of the TV, with either direct chordal attachment to the septum or through the so-called Lancisi conal papillary muscle (PM). The TV leaflets are thinner, more translucent and more fragile compared to the MV (Figures 3, 6).

### Interventional perspectives

Due to the different tissue property and characteristics, the chance of damaging or tearing the TV leaflets is higher compared to the MV. This has to be taken into consideration in case of leaflet repair, as MitraClip in tricuspid position.

## Subvalvular apparatus

The subvalvular apparatus of the MV and TV is similar and consists of two different structures with different characteristics: the papillary muscles (contractile function) and the chordae tendinae (elastic function).

### Papillary muscles

#### Mitral

**Figure 7:**
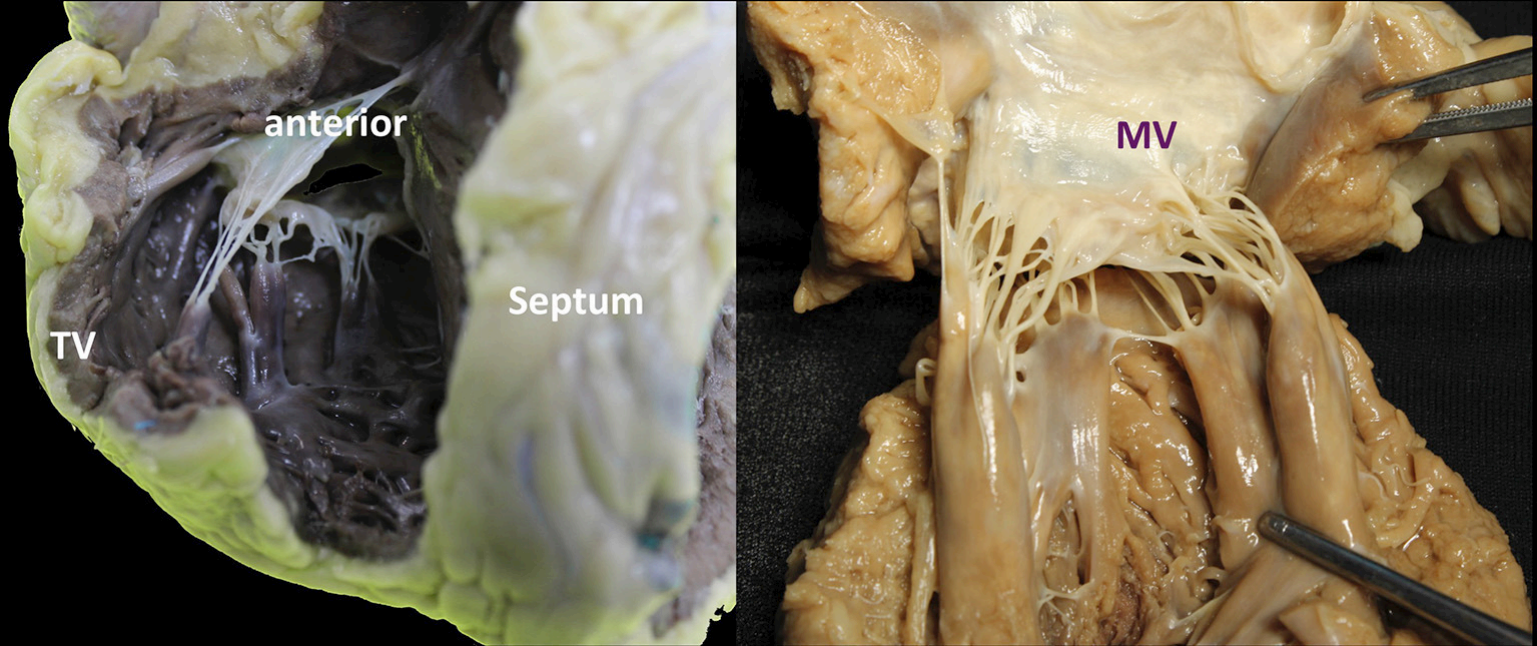
Comparison of mitral and tricuspid PMs. **(A,B**) Each group of papillary muscles comprises either a single bulky papillary with multiple heads or otherwise several thinner papillary muscles from which arise the numerous chordae attaching to the leaflets. They are implanted on the muscular wall of the left ventricle at a junction situated ~1/3 from the apex and 2/3 from the annulus. The position is varying little: the anterolateral PM is implanted at the junction between the septum and the posterior wall of the ventricle. The postero-medial PM is inserted on the lateral wall of the ventricle. The length of the PMs is variable, ranging from 2 to 5 cm. Importantly, no PMs attach to the left side of the ventricular septum.

The mitral PMs, which insert on the left ventricular (LV) free wall, are usually organized into two groups, which are the posteromedial and the anterolateral, situated just below the corresponding commissures (Figure 7). Not rarely, a third intermediate PM is found implanted between them, providing the chordae to A2 or P2 segments. Apical displacement of the posteromedial PM secondary to lateral myocardial infarction is the most frequent mechanism to underline asymmetrical tethering and functional MR post-infarction chronic ischemic heart disease.

#### Tricuspid

**Figure 8:**
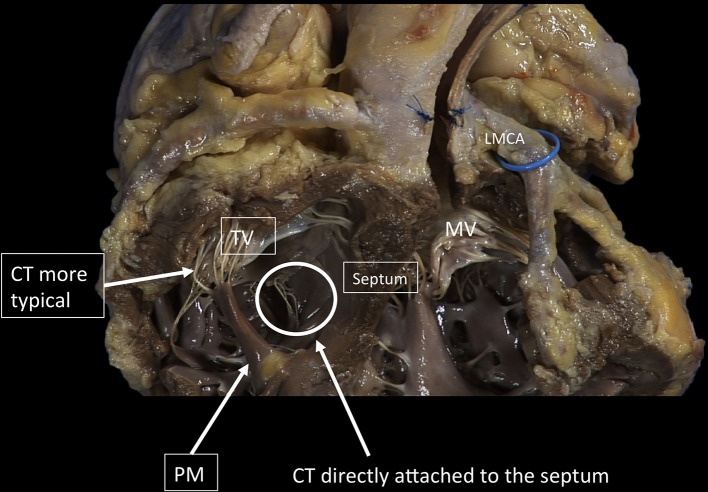
The TV has a more complex chordal structure in comparison to MV. The chordae can be attached directly to the RV wall and septum, differently from the LV. On average, a number of 25 chordae inserts into the TV. The chordal system is hierarchical similar to the left one, dividing the types of chordae in basal, intermediary or secondary chordae and the marginal chordae, attaching to the free edges. The attachment of the marginal or primary chordae is usually single on the right, without bifurcating or trifurcating close to the edge and originating from various levels of the papillary muscles. Even if thinner, the chordae of the TV commissures are trifurcated with a characteristic fan-like disposition. CT, Chordae tendineae; PM, papillary muscle; MV, mitral valve; TV, tricuspid valve. LMCA, left main coronary artery (light blue circle). Courtesy of Dr. M. Reisman, University of Washington.

The tricuspid PMs are inserted on the right ventricular (RV) wall and usually organized into three groups: anterior, posterior and septal (Figures 7, 8). The anterior PM is the dominant and is implanted on the anterior wall of the RV, near the apex, fusing with the moderator band. The chordae tendineae extend from the free margin to the PM. Three types can be described: the basal chordae (tertiary), the intermediary chordae (secondary) and the marginal chordae (primary), which are the most represented (8). Basically, having the TV three leaflets, with the posterior often divided in further scallops, it presents a more complex chordal structure in comparison to MV (9).

### Interventional perspectives

The main interventional issue related to the subvalvular apparatus is the risk of impingement of any device in the chordal apparatus, once the valve is crossed. Similarly in both MV and TV, the risk is higher in the commissural region, in which the density of chordae is the maximum, while the middle of the valve is chordae-free zone. This is particularly true for leaflet repair devices delivered antegrade, typically with the MitraClip. In presence of a commissural lesion, the risk of clip impingement is particularly high and can lead to impossibility to retrieve the device or chordal rupture with consequent worsening of the regurgitation. In TV the commissures are almost invariably the first therapeutic target (usually the antero-septal): a first clip is implanted close to the commissure, where the coaptation deficit is minimum, in order to approximate the leaflets, and may facilitate the implantation of further clips on the coaptation line. Since the only location that allows leaflet grasping is at the real commissure, risk of impingement or chordal injury is present in tricuspid clipping procedures. Similarly to the leaflet, also the chordal tissue of the TV is thinner and more fragile compared to MV, and this may increase the risk of damage, especially in case of multiple grasping attempts or in case of chordal impingement.

## Left ventricle, right ventricle and outflow tracts

### Anatomical description

#### Left ventricle

**Figure 9:**
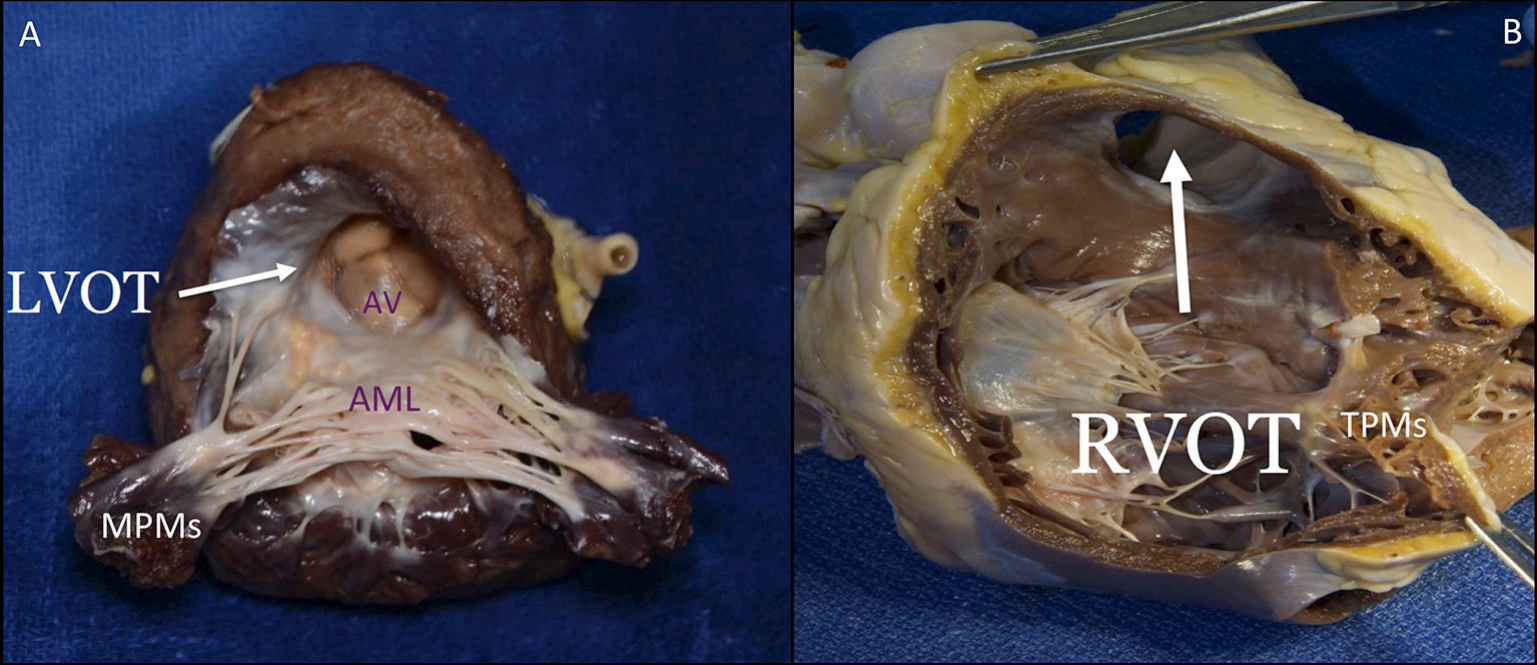
The septal surface of the LV may be considered to have a sinus portion, most of which is trabeculated, and a smooth outlet (LVOT) portion **(A)**. The part of the sinus portion of the septum immediately beneath the mitral valve may be termed the inlet septum, and the rest of the sinus portion, the trabecular septum. The LVOT lies in front and to the right of the anterior mitral leaflet, corresponding to the inlet portion on the right ventricular side of the septum, and includes the atrioventricular septum. On the right side, the septal leaflet is the only one attached to the septum, but it leaves the RVOT free **(B)**. The lowermost of the small septal muscles attaches posterior to the trabecula septomarginalis and the uppermost, called the medial or conal PM (muscle of Lancisi), to the posterior limb of the septal band. The septal PM is almost not affected by tethering in case of RV dilatation. LVOT, left ventricular outflow tract; AV, aortic valve; AML, anterior mitral leaflet; MPMs, mitral papillary muscles; RVOT, right ventricular outflow tract; TPMs, tricuspid papillary muscles. This image has been adapted from Taramasso et al. (1).

The LV consists of a larger sinus portion, which supports the MV and includes the apex, and a much smaller outlet (outflow) portion beneath the aortic semilunar valve. Contrary to the RV, the inlet and outlet valves of the LV lie juxtaposed within its base, and inflow and outflow portions are separated by a curtain represented by the anterior MV leaflet (Figure 9). The LV trabeculations are characteristically fine compared with those in the RV (10).

#### Right ventricle

**Figure 10:**
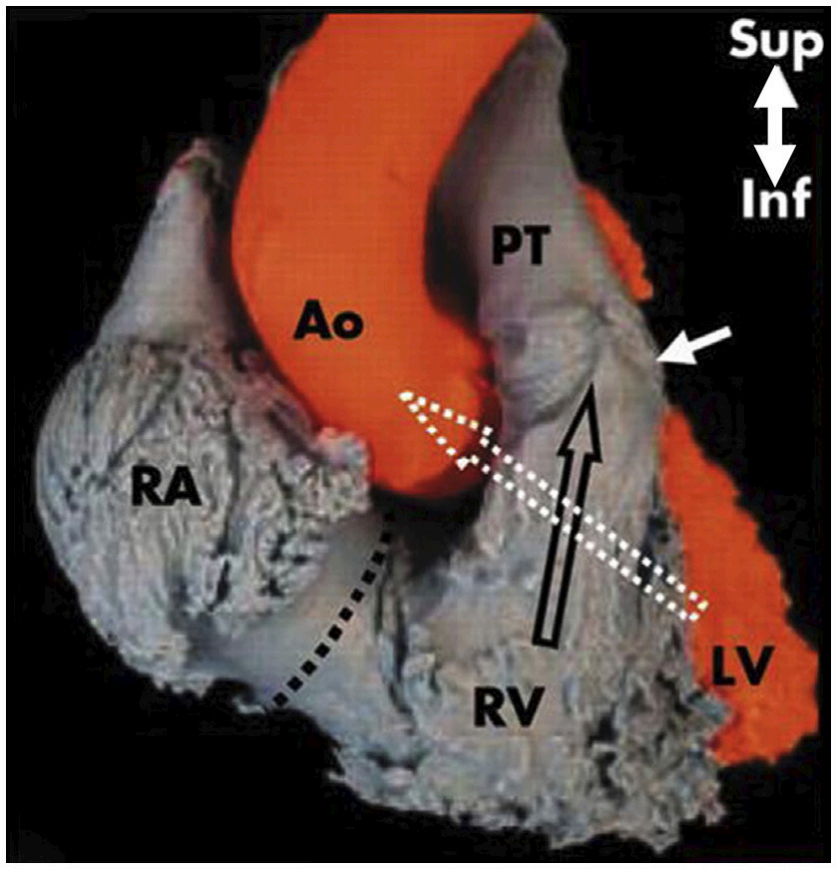
The right outlet portion of the ventricular septum is smooth and has three components. The largest is the infundibular septum, which separates the pulmonary valve (with arrow) from the aortic and TV. A second part of the outlet portion of the septum is the anterior extension of the trabecular septomarginalis (septal band). A third small, very anterior portion is a narrow extension superior to the trabecular septum. The axes of the right and left ventricular outflow tracts differ significantly. That of the RV is almost vertically oriented, whereas that of the left ventricle angles sharply to the right, a characteristic criss-cross feature (white and black empty arrows), visible under fluoroscopy in the left anterior oblique projection and in the parasternal long axis view by two-dimensional echocardiography. RA, right atrium; Ao, Aorta; RV, right ventricle; PT, pulmonary trunk; LV, left ventricle. Modified from Ho et al. (12).

The RV has a large sinus portion that surrounds and supports the TV (inlet portion) and includes the apex and an infundibulum (outlet portion) that supports the pulmonic valve. The inlet and outlet valves of the RV, opposite to the aortic and MV, are thus widely separated by the “Crista ventricularis” (11), minimizing any risk of right ventricular outflow tract obstruction (RVOT). The entire sinus portion of the RV and most of the infundibulum (both free wall and septum) are coarsely trabeculated (12). The conduction system (bundle of His) perforates the central fibrous body closer to the RV side, therefore the possibility to damage this structure from the left, in comparison to the right side (Figure 10), is very remote.

### Interventional perspectives

The close relationship between the anterior mitral leaflet, the aortic valve and the left ventricular outflow tract (LVOT) has important consequences: the implantation of a transcatheter heart valve inside the native or repaired MV forces the anterior leaflet in an “open position,” that may encroach on the LVOT. This septal displacement of the anterior mitral leaflet is exaggerated when the aortic and mitral annular planes are acutely angulated, when the interventricular septum is hypertrophic and bulges toward the LVOT, in presence of an elongated leaflet, and when the valve implant extends or flares into the LV. On the contrary, the marked separation between the TV and the pulmonary valve by the Crista supraventricularis and the wide-open angle between them make the risk of RVOT obstruction really low with any type of tricuspid device, in any anatomical context (Figures 9, 10). While transapical LV access is frequently used for aortic and MV procedures, apical RV access presents several issues. The thin and trabeculated RV wall makes this approach potentially risky, especially in the context of RV dilatation and dysfunction associated to functional TR.

## Conclusions

With the fast development of transcatheter TV therapies, physicians are facing up with a new challenging anatomical scenario. A deep understanding of the anatomy of the TV and of the right heart chambers, and their differences compared to the left-heart, is fundamental to improve safety and efficacy. The specific anatomical features of the TV, the low quality of intraprocedural TEE guidance and the absence of a standardized nomenclature remain major open issues to be addressed in TV intervention.

## Author contributions

AP and MT wrote the manuscript. MZ, MR, and FM supervised, contributed with specific iconography and revised critically the paper.

### Conflict of interest statement

MT is consultant for Abbott Vascular and 4Tech; FM is consultant for Abbott Vascular, Edwards Lifesciences, Medtronic and co-founder of 4Tech. The remaining authors declare that the research was conducted in the absence of any commercial or financial relationships that could be construed as a potential conflict of interest.

## Abbreviations

AV: atrio-ventricular
TAVI: transcatheter aortic valve implantation
MV: mitral valve
TV: tricuspid valve
LA: left atrium
RA: right atrium
LAA: left atrial appendage
RAA: right atrial appendage
TEE: transesophageal echocardiography
TTE: trans-thoracic echocardiography
ICE: intra-cardiac echocardiography
LV: left ventricle
RV: right ventricle
PM: papillary muscle
LVOT: left ventricle outflow tract
RVOT: right ventricle outflow tract.
